# A multisite randomized controlled trial of VA integrated and enhanced referral behavioral health models on alcohol misuse in older male veterans

**DOI:** 10.1186/1940-0640-10-S1-A73

**Published:** 2015-02-20

**Authors:** Nikki R Wooten, Abbas S Tavakoli, Marlene B Al-Barwani, Naomi A Thomas, Selina H McKinney, Anna M Scheyett, Kelly M Kaminski, Alyssia C Woods, Sue E Levkoff

**Affiliations:** 1 College of Social Work, University of South Carolina, Columbia, SC, 29208, USA; 2 District of Columbia Army National Guard, Washington, DC, 20003, USA; 3 William Bryan Jennings Dorn Veteran Affairs Medical Center, Columbia, SC, 29209, USA; 4 College of Nursing, University of South Carolina, Columbia, SC, 29208, USA

## Background

Despite frequent primary care visits, older veterans may have unmet behavioral health needs regarding alcohol misuse. Identification of alcohol misuse and referral of older veterans seen in primary care to behavioral health care can decrease at-risk drinking and assist in determining effective interventions for older veterans with problem drinking. This study examined the effect of integrated and enhanced referral behavioral health models on alcohol misuse among older veterans.

## Methods

This was a secondary analysis of data collected at five Department of Veteran Affairs (VA) Medical Centers as part of the Primary Care Research in Substance Abuse and Mental Health for Elderly study, a multisite randomized controlled trial. The sample included 1222 older male veterans who were randomly assigned to integrated or enhanced referral behavioral health care at VA primary care clinics with 6 to 12-month follow-up. Brief alcohol treatment was provided to at-risk drinkers by behavioral health providers either co-located within primary care clinics (integrated treatment model) or a separate location offering transportation assistance and coordinated care between behavioral health providers and primary care physicians (enhanced referral model). Alcohol misuse included both at-risk drinking for older adults (> 1 drink daily) and problem drinking assessed using the Short Michigan Alcoholism Screening Test-Geriatric Version (problem drinking ≥ 3 affirmative responses). Both behavioral health models required a minimum of one treatment session, with no maximum number of sessions over a 6-month period. After 6 months, attendance at treatment sessions was voluntary.

## Results

At baseline, 35.8 percent (n = 438) reported at-risk drinking and of those, 46.1 percent (n = 200) reported problem drinking. At 6 months, 21.1 percent (n = 234) reported at-risk drinking and of those, 41.4 percent (n = 150) reported problem drinking. At 12 months, only 36.3 percent (n = 89) reported problem drinking. Bivariate analyses revealed no significant differences between behavioral health models in at-risk and problem drinking over time. Generalized linear mixed modeling revealed treatment and time effects were significant for at-risk drinking after controlling for VA site, race, education, employment, and depressive symptoms. Treatment and time effects were significant for problem drinking after controlling for VA site, race, and depressive symptoms. The treatment x time effect was non-significant for at-risk and problem drinking.

## Conclusions

Both integrated and enhanced referral behavioral health models were effective in reducing at-risk drinking among older male veterans seen in VA primary care clinics with follow-up over 12 months. However, the integrated model was more effective than the enhanced model in reducing problem drinking. Screening and brief intervention for alcohol misuse in primary care can be cost effective by reducing service utilization and potentially result in a medical cost offset. Based on primary care protocols, VA health administrators and policymakers should determine which treatment model is best for their patients, providers, and infrastructure. Future research should examine treatment processes of both behavioral models to determine their effect on alcohol misuse in older veterans.

